# A guide to the contained use of plant virus infectious clones

**DOI:** 10.1111/pbi.12876

**Published:** 2018-02-06

**Authors:** Helen C. Brewer, Diane L. Hird, Andy M. Bailey, Susan E. Seal, Gary D. Foster

**Affiliations:** ^1^ School of Biological Sciences University of Bristol Bristol UK; ^2^ Natural Resources Institute University of Greenwich Chatham Maritime Kent UK

**Keywords:** plant virus, infectious clone, biosafety level, containment, GMO

## Abstract

Plant virus infectious clones are important tools with wide‐ranging applications in different areas of biology and medicine. Their uses in plant pathology include the study of plant–virus interactions, and screening of germplasm as part of prebreeding programmes for virus resistance. They can also be modified to induce transient plant gene silencing (Virus Induced Gene Silencing – VIGS) and as expression vectors for plant or exogenous proteins, with applications in both plant pathology and more generally for the study of plant gene function. Plant viruses are also increasingly being investigated as expression vectors for *in planta* production of pharmaceutical products, known as molecular farming. However, plant virus infectious clones may pose a risk to the environment due to their ability to reconstitute fully functional, transmissible viruses. These risks arise from both their inherent pathogenicity and the effect of any introduced genetic modifications. Effective containment measures are therefore required. There has been no single comprehensive review of the biosafety considerations for the contained use of genetically modified plant viruses, despite their increasing importance across many biological fields. This review therefore explores the biosafety considerations for working with genetically modified plant viruses in contained environments, with focus on plant growth facilities. It includes regulatory frameworks, risk assessment, assignment of biosafety levels, facility features and working practices. The review is based on international guidance together with information provided by plant virus researchers.

## Introduction

Plant viruses are important not only because of the threat they pose to global crop production, but also due to their use as research tools in molecular plant pathology and other areas of biotechnology (Rybicki, 2015; Scholthof *et al*., 2011). Much of this success hinges on the ability to maintain, replicate and modify plant viral genomes in the form of infectious clones (ICs).

Infectious clones consist of plant viral genome material maintained as cDNA or DNA templates within bacterial plasmids, usually in *Escherichia coli*, affording ease of *in vitro* propagation, storage and genetic manipulation. These plasmids can then be used for *in vitro* transcription of viral RNA for direct inoculation of plants (for RNA viruses) or transformed into *Agrobacterium tumefaciens* for inoculation of plants by agroinfiltration (both RNA and DNA viruses) (Dagless *et al*., 1997; Feng *et al*., 2016; Peyret and Lomonossoff, 2015; Zarzyńska‐Nowak *et al*., 2017). This then results in infection and the reconstitution of fully functional self‐replicating virus genomes within the plant host.

Plant virus ICs have a broad range of applications. Within the field of plant pathology, the creation of infectious clones containing wild‐type viral genomes provides a ready source of inoculum for disease resistance screening of different host genotypes. This negates the need to maintain and passage the virus within plants and provides an alternative to laborious or inefficient inoculation techniques such as grafting or infection by insect vectors. ICs also provide a genetically uniform source of inoculum, removing obstacles caused by genetically variable viral populations or mutations occurring during plant passage.

Examples include the use of Cassava mosaic virus ICs to screen transgenic cassava for virus resistance, aiding the development of cassava lines with resistance to the devastating diseases caused by these viruses (Beyene *et al*., 2016; Kuria *et al*., 2017). In addition, infectious clones can also be used to study the host response at a molecular level. For example, Tomato chlorotic mottle virus infectious clones were used to compare the proteomes of resistant and susceptible near‐isogenic tomato lines following infection (Carmo *et al*., 2017).

The use of infectious clones allows for modification of the viral genome prior to inoculation, with various applications. For example, Martin and Rybicki (2002) identified the pathogenicity determinants of a highly pathogenic maize streak virus strain by systematically ‘swapping out’ sections of its genome with those from less pathogenic strains, creating chimaeric infectious clones. Duff‐Farrier *et al*. (2015, 2016) used chimaeric infectious clones of Pepino mosaic virus to identify the pathogenicity determinants of different virus strains within various plant hosts. Similarly, Harper *et al*. (2016) identified the genetic determinants of Citrus tristeza virus transmissibility by aphids by systematically substituting sequences from a highly transmissible strain into a poorly transmissible strain.

Chimaeric ICs have also been used to study the constraints of plant virus recombination in an evolutionary context (Martin *et al*., 2005). Tagging of plant viral genomes with fluorescent reporter genes is also widely used both to track viral movement *in planta* and to elucidate the function of specific viral proteins. For example, Martinez and Daros (2014) used fluorescent tagging of the Tobacco etch virus P1 protein to track its subcellular localization and interaction with host proteins.

In addition to their role in the study of plant–virus interactions, modified plant viruses can also be used to trigger silencing of host genes (known as virus induced gene silencing or VIGS) without the need for stable plant transgenesis (Lange *et al*., 2013; Lee *et al*., 2012). VIGS involves modifying infectious clones to contain a short sequence of the host gene of interest, resulting in post‐transcriptional silencing of the gene as part of the plant's own defence response following virus infection. For example, modified Barley stripe mosaic virus is widely used to silence genes in polyploid cereals such as wheat. Beyond silencing plant genes, VIGS can also be used to silence the genes of other plant pathogens during co‐infection, further aiding the study of plant–pathogen interactions and exploring gene function in pathogens not amenable to modification (reviewed in Lee *et al*., 2012).

However, the use of ICs is no longer the preserve of plant molecular biologists and pathologists. Modified plant viruses are also increasingly being used in other disciplines as expression vectors for heterologous proteins, particularly with biopharmaceutical and industrial applications (known as molecular farming). Such applications have been extensively reviewed elsewhere (Canizares *et al*., 2005; Daniell *et al*., 2009; Gleba *et al*., 2007; Hefferon, 2012, 2014; Marsian and Lomonossoff, 2016; Nagyová and Šubr, 2007; Plchova *et al*., 2017; Pogue *et al*., 2002; Saunders and Lomonossoff, 2013; Steele *et al*., 2017).

Briefly, plants can be used as living ‘factories’ for foreign protein production and these systems are less costly than other eukaryotic bioreactors. The use of ICs to transiently express the gene of interest *in planta* is often favoured over stable transgenesis, due to the ease and speed with which viral genomes can be modified compared to those of plants, the high levels of protein production achieved and the potential for use in a broad range of hosts. Such systems show promise for the generation of vaccines, antigens, hormones, therapeutic antibodies, industrial biopolymers and bio‐nanoparticles.

There has been no single comprehensive review of the biosafety considerations for the contained use of genetically modified plant viruses, despite their increasing importance across many biological fields. This review therefore explores the biosafety considerations for working with IC‐derived plant viruses in contained environments, with focus on plant growth facilities. It includes regulatory frameworks, risk assessment, assignment of biosafety levels, facility features and working practices. The review is based on international guidance together with information provided by plant virus researchers.

## Biosafety and plant virus ICs

Plant virus ICs are powerful tools with applications across multiple scientific fields from plant pathology to biopharmaceutical production. However, their use carries potential environmental risks and is therefore subject to containment and regulation. Once inside a plant cell, the viral genome is translated or released, leading to reconstitution of a fully functional virus that is then capable of replication and potentially of spread within the plant and transmission to other plants. In addition, while unmodified plant viruses do not pose a risk to human health (Nikitin *et al*., 2016), they could potentially be used as expression vectors for genes encoding proteins known to have some degree of human allergenicity or toxicity, with potential health implications for researchers and the wider population (NIH 2013; Wagner *et al*., 2004). Despite this, there is no single source of guidance tailored specifically towards effective containment of genetically modified plant viruses. The relevant information is scattered across numerous documents from multiple countries and therefore not easily accessible to researchers. In this review, we therefore consolidate this information to highlight the biosafety considerations for the contained use of plant viruses. We also review individual risk assessments and protocols provided by researchers currently working with plant virus ICs in Europe and the United States. We focus predominantly on mitigation of environmental risks from the use of ICs in plant growth facilities. We also highlight complexities in the regulatory and approval process for working with plant virus infectious clones, using the UK framework as an example.

## Risk groups and biosafety levels

The World Health Organization Laboratory Biosafety Manual (WHO 2004) provides international guidance for working with disease‐causing and/or genetically modified microorganisms. It sets out four risk groups for these microorganisms based on the likelihood and impact of release or exposure, ranging from 1 being low to 4 the highest risk. It then sets out four corresponding laboratory biosafety levels (BSLs), each with defined requirements for laboratory design and operating procedures (WHO 2004). This guidance has been adopted globally and translated into broadly comparable national regulations and guidance (Tian and Zheng, 2014).

However, the WHO Laboratory Biosafety Manual is largely focussed on containment of pathogens of humans and animals in a laboratory setting and not the environmental risks posed by contained use of genetically modified plants or plant pathogens. There is no equivalent international level guidance for plant containment facilities.

Many countries are signed up to international treaties related to plant biosafety, namely the Cartagena Biosafety Protocol (https://bch.cbd.int/protocol) and the International Plant Protection Convention (https://www.ippc.int). However, the former is primarily concerned with the deliberate release of genetically modified plants, while the latter aims to control the spread of plant pests and pathogens already present in nature. Neither currently sets out guidance on contained use.

It has therefore fallen to individual countries to build upon the WHO laboratory guidelines, to create guidance applicable to the contained use of plants and plant pathogens (including plant virus ICs) within plant growth facilities, with consideration for the environmental risks posed. This has resulted in the creation of country‐specific but broadly comparable guidance for the containment of GM plants and plant pathogens, including the adoption of biosafety levels specific to plants.

### Biosafety levels for plants

The United States was the first country to build upon the laboratory biosafety levels set out by WHO to set out four biosafety levels for plants (BLPs) as described in the USA National Institutes of Health biosafety guidelines (NIH 2013). These are summarized below:

- BLP1: Designed for containment of experiments that pose no recognizable or predictable risk to the environment in the event of accidental release.
- BLP2: Appropriate for experiments where there is a possibility of survival and dissemination of plant‐related material in the event of accidental release, but where this would have a minimal biological impact.
- BLP3: Designed to minimize or prevent spread or release of organisms that have the potential for significant environmental harm. It is also appropriate for containment of plants or associated microbes producing vertebrate toxins.
- BLP4: The highest containment level may be required for containment of certain exotic plant pathogens, including viruses in the presence of their arthropod vector.

The NIH (USA) also describes biological containment methods (such as removal of flowering plant parts) which can be used to reduce the biosafety level requirement in some instances. Further examples of biological containment of plant virus ICs are described under ‘Biological Containment’ later in this review. The NIH guidelines are expanded upon in ‘A practical guide to containment: Plant biosafety in research greenhouses’ (Adair and Irwin, 2008). The first edition of this manual (Traynor *et al*., 2001) along with the NIH guidelines has been used as a reference point for several other countries when developing their own guidance or legislation for plant containment (Department of Agriculture (South Africa), 2004; UNCST, 2007; Adair and Irwin, 2008; Replublic of Kenya, 2009; Tanzania, 2012; Australian Government, 2012). This has resulted in broadly comparable guidelines and plant biosafety level designations across the globe, with countries such as South Africa adopting the USA BLP designations *ad verbum* (Department of Agriculture, South Africa, 2004).

An equivalent framework of plant biosafety levels has also been adopted across the European Union, enacted in EU directive 2009/41/EC. As such, the Health and Safety Executive in the United Kingdom also sets out four biosafety levels (BSLs) for plant growth facilities (HSE, 2004). However, unlike the NIH, the UK HSE does not state the purpose of each BSL in summary form. Rather, assignment of the BSL for an activity involving plant‐associated genetically modified microorganisms (GMMs) is based on a detailed risk assessment (see ‘Risk Assessment’ below).

BSL4 is not represented in the UK guidance as no such plant growth facility currently exists in the United Kingdom. BSL1 is only suitable for activities with ‘no or negligible risk’; therefore, *in planta* work involving plant virus ICs is likely to be carried out at BSL2 or BSL3. The building, equipment and operational requirements for these BSLs are summarized in Table 1, with differences in requirements between BSLs highlighted. The key differences between BSL2 and BSL3 are the requirement for negative pressure and air filtering, sealed flooring and waste treatment within the facility at BSL3.

**Table 1:** Requirements for plant growth facilities operating at Biosafety (Containment) levels 2 and 3. Y – Required. N – Not required. Y/N – Required where and to the extent that the risk assessment shows it is required. Differences between containment levels 2 and 3 are highlighted in yellow. Modified from the UK SACGM Compendium of Guidance Part 4 (HSE 2004)

Containment measures	Containment Level 2	Containment level 3
Building		
Permanent structure	Y	Y
Laboratory suite: isolation	N	Y
Laboratory: sealable for fumigation	N	Y
Equipment		
Impervious, easy to clean surfaces	Y – bench	Y – bench and floor
Entry via an airlock or a separate room with two interlocking doors	Y/N	Y/N
Negative pressure relative to immediate surroundings	Y/N	Y
HEPA filtered extract air	N	Y
Microbiological safety cabinet/enclosure	Y/N	Y
Autoclave	Y – in building	Y – in laboratory suite
Control of contaminated run‐off water	Y – to minimize run‐off	Y– to prevent run‐off
System of Work		
Restricted Access	Y	Y
Specific measures to control aerosol dissemination	Y – to minimize	Y – to prevent
Shower	N	Y/N
Protective clothing	Y	Y
Protective footwear	N	Y/N
Gloves	Y/N	Y
Effective control of disease vectors which could disseminate the GMM	Y	Y
Effective control of plant material which could disseminate the GMM	Y – to minimize dissemination	Y – to prevent dissemination
Procedures for transfer of living material between facilities shall control dissemination of GMMs	Y – to minimize dissemination	Y – to prevent dissemination
Specified disinfection procedures	Y	Y
Waste		
Inactivation of GMMs in effluent from hand‐washing sinks and showers and similar effluents	N	Y/N
Inactivation of GMMs in contaminated materials and waste	Y – by validated means	Y – by validated means, with waste inactivated in the laboratory suite
Laboratory to contain its own equipment	N	Y – so far as is reasonably practicable
An observation window or alternative is to be present so that occupants can be seen	Y/N	Y
Safe storage of GMMs	Y	Y
Written records of staff training	Y/N	Y

Some countries such as Canada have slightly different minimum requirements and numbering systems for each biosafety level; Canadian Plant Pest Containment (PPC) levels 1‐3 roughly correspond to UK‐BSL/USA‐BLP2‐4 (Canadian Food Inspection Agency 2014).

## Risk assessment

A risk assessment is necessary for activities involving pathogenic microorganisms, including plant viruses. The aim of the assessment is to identify and define risks posed to the environment and to human health and identify control measures required to mitigate these risks. Some national competent authorities provide step‐by‐step guidance on performing risk assessments for working with plant‐associated GMMs. For example, in the United Kingdom, the HSE require the use of a risk determination matrix, which considers the likelihood of release against the consequences should a release occur (Table 2). The risk is considered high when there is a high likelihood of release along with severe consequences in the event of a release. Conversely, if both the likelihood and consequences of a release are negligible, then the risk can be considered as effectively zero (HSE, 2004). Containment measures must be selected that reduce the overall risk to low or effectively zero. Other countries such as Canada also use risk determination matrices to inform decisions regarding the biosafety measures required. Canada's matrix is based on the risk of escape and establishment in the absence of physical containment and assigns a required biosafety level accordingly. However, the physical attributes of the facility must be adequate for containment regardless of risk posed (Canadian Food Inspection Agency 2014).

**Table 2:** Risk determination matrix for assessing the level of risk posed by a contained use activity involving GM plant viruses, modified from the UK SACGM Compendium of Guidance Part 4 (HSE, 2004). The biosafety level and containment measures selected for the activity must be sufficient to reduce the risk to low or effectively zero

		Likelihood of Hazard			
		High	Medium	Low	Negligible
Consequence of Hazard	Severe	High	High	Medium	Effectively Zero
	Modest	High	Medium	Medium/Low	Effectively zero
	Minor	Medium/low	Low	Low	Effectively Zero
	Negligible	Effectively Zero	Effectively Zero	Effectively Zero	Effectively Zero

A risk assessment needs to consider many factors, such as whether the virus is indigenous, its host range, effects of genetic modification, the presence of hosts or vectors and interactions with other organisms within or around the containment facility. In addition to the nature of the virus, the nature of the activity should also be considered, such as experiment duration and scale (HSE, 2004; Department of Agriculture (South Africa), 2004; Adair and Irwin, 2008; Canadian Food Inspection Agency 2014). The following sections highlight key considerations for plant virus risk assessments:

### Effects of genetic modification

In the case of genetically modified plant virus ICs, the risk assessment needs to consider not only the inherent risks of the virus but also how the risks might be altered by its modification (Department of Agriculture (South Africa) 2004; Phillipson and Weekes, 2005; HSE, 2004). For example, an otherwise low‐risk virus modified to express a fungal virulence factor may pose and environmental risk by making infected plants more susceptible to fungal pathogens. Similarly, a virus carrying a construct to silence a trait involved in crop yield would present a higher risk to nearby host crops than the unmodified virus (Lee *et al*., 2012). Genetic modifications may also impact the host range, survival and transmission of the virus or result in loss of host resistance. For example, introduction of a coat protein mutation in *Pepino mosaic virus* breaks Rx‐mediated resistance in solanaceous hosts (Duff‐Farrier *et al*., 2016) and so would be a higher risk than ICs that were not able to overcome such host resistance.

### Interactions with other organisms

There may be additional risks posed by other organisms contained in close proximity to the plant virus or indeed be part of the same experimental system (Adair and Irwin, 2008). These include GM or exotic plants, insect vectors and other infectious agents such as *Agrobacterium tumefaciens* that may have been used to introduce the viral genome into the host plant. Consideration should be given not only to the inherent risks of these organisms but also to the potential interaction between them and the infectious clone, aiding dissemination and transmission. Plant growth facilities are often used by multiple researchers for various projects, which may have different containment requirements, and operational practices need to reflect this.

### Establishment in the environment

Risks of establishment and survival of a plant virus depend not only on the virus and any modifications, but also on the immediate environment. Viruses are unlikely to persist in an environment where their host and/or vector are absent. The risk may also be considered lower in countries where the plant host is present but not economically important or widely cultivated. Therefore, ICs that pose a prohibitively high risk in one country or region may be used with relatively low risk in another. However, the possibility of unknown hosts or vectors that would allow survival and establishment should always be carefully considered, as should the ability of the virus to adapt to infect new hosts or vectors.

### Consideration of socioeconomic factors

When assessing the impact of virus escape and establishment, it is important not only to consider the impact on host plants, such as crops, but also the capacity of a country to identify, respond to and mitigate a containment breach. In the case of crops, the potential downstream impact on farmer livelihood must be estimated. More economically developed countries with greater food security might be less impacted by a disease outbreak than less economically developed countries with a small number of staple subsistence crops, diseases of which could result in loss of livelihood and even famine (Thresh and Hillocks, 2003). These countries may also be less well equipped to contain disease spread, exacerbated by mixed cropping systems and year‐round availability of hosts.

### Derogations from BSL requirements

It is important to identify the most appropriate measures for virus containment on a case by case basis and then assign the minimum Biosafety Level (BSL) that ensures these measures are implemented, rather than simply applying generic containment measures based on BSL, which may not be appropriate for containment (HSE, 2004). The risk assessment may therefore identify extra containment measures which are not specified for a given BSL, but also cases where some features of the designated BSL are not appropriate or beneficial for containment. This may be because they are superfluous to requirements, or indeed because they actively impede containment measures. In this situation, the researchers may apply for derogations from the BSL. A common derogation is the lack of microbiological safety cabinets at BSL3, as these are inappropriate for *in planta* work. Other examples may be specific to plant virus work; for example, the negative pressure gradient normally required at BSL3 could promote the ingress of insect vectors which could spread the virus throughout the facility. HEPA filters, for example, are required at BSL3; however, the use of a G4 filter may be more appropriate for containing pollen‐borne viruses (Adair and Irwin, 2008).

## Common shortcomings of risk assessments for genetically modified plant viruses

Research commissioned by HSE (UK) into the containment of genetically modified plant viruses has identified several common shortcomings and inconsistencies in the risk assessment process (Phillipson and Weekes, 2005). The UK Scientific Advisory Committee on Genetic Modification (SACGM) compendium of guidance also provides an example risk assessment for working with GM plant viruses, highlighting some of the details that researchers may fail to include when performing a risk assessment (HSE, 2004). Common shortcomings include the reliance on expert opinion rather than empirical data, as well as specific risk factors being described qualitatively rather than quantitatively. This is particularly true of the assessment of risks related to the stability of genetic modifications, the presence of hosts and/or vectors and the potential for spread of mechanically transmitted viruses as outlined below:

### Stability of genetic modification

Researchers often state that, should a modified plant virus IC be released, the nature of virus replication means that the genetic insertion is likely to be lost after a number of rounds of replication, rendering the virus equivalent to wild type, as observed for the frequently used TMV expression system (Donson *et al*., 1991; Kohl *et al*., 2006; Rabindran and Dawson, 2001; Varsani *et al*., 2006). This argument is used to qualify the low risks posed by multiple species of genetically modified indigenous viruses. However, the likelihood of the insert persisting will depend on the virus in question and the size and nature of the insert, and so should be quantified on a case by case basis (Hefferon, 2014; Phillipson and Weekes, 2005).

Conversely, and particularly in the case of molecular farming, researchers use genetic modification to ‘disable’ viruses carrying exogenous proteins, for example by removing genes required for dissemination or transmission (Gleba *et al*., 2007; Hefferon, 2014). Their evaluation of risk relies on the assumption that this modification is stable and would persist should the infectious clone be released into the environment, without acknowledging that the virus could be rendered fully infective via viral recombination, whilst maintaining the genes for exogenous protein production (Phillipson and Weekes, 2005).

### Identification of hosts and vectors

The absence of native hosts or vectors in the environment surrounding the containment facility is often used as a basis for low risk, especially in the case of nonindigenous viruses. However, lack of evidence of a known vector in the region where work is to be done using a nonindigenous virus does not mean that no vector exists. This also applies to host range, as there may be unidentified hosts present in the local environment. Where possible preliminary transmission studies should be done to ascertain risk of transmission by native insect pests and/or to native plant hosts (e.g. see Phillipson and Weekes, 2005).

### Mechanical transmission

Mechanically transmitted viruses may be inadvertently spread throughout a containment facility by physical contact between plants, or contact between plants and contaminated equipment such as watering cans or gloves. Equivalent risk of accidental spread between plants is often apportioned to multiple species of mechanically transferred viruses, without quantification of the rates of transfer. However, Phillipson and Weekes (2005) found significantly different rates of mechanical transmission between two commonly used plant viruses: Tobacco mosaic virus and Potato Virus X. It cannot be assumed that all mechanically transmitted viruses have an equivalent risk of accidental spread.

It is therefore important that where possible, risk assessments are based on established evidence and that a precautionary approach is adopted where there is an element of uncertainty.

## Dual use and deliberate release

In addition to the risks posed by unintentional release of a plant virus from a containment facility, it is also important to consider the potential for deliberate release or malicious use of plant virus infectious clones, known as dual‐use risk. While biological warfare is generally associated with human disease agents such as anthrax, there is the potential for bioterrorism using plant pathogens, which could have devastating effects on food security. It has been proposed that targeting crops may be simpler and less technologically challenging than biological warfare against humans (Madden and Van Den Bosch, 2002; Wheelis *et al*., 2002; Whitby, 2001). The USA Centers for Disease Control (CDC) maintain a list of ‘select agents’ that are considered a bioterrorism risk and require additional containment and regulation. This list includes some plant pathogens but does not currently include any plant viruses. However, there is concern that molecular farming using modified plant viruses has the potential for dual use due to the ability to produce large quantities of human toxins *in planta* (Federation of American Scientists 2011). In the United States, there is a statutory requirement to declare research with a dual‐use risk but this requires that the risk has been identified; plant researchers may not consider that their research outcome could have a dual use. It is therefore important to consider the possibility of dual use during a risk assessment, to liaise with the appropriate competent authorities and to instigate proportionate containment and security measures to guard against misappropriation.

## Containment methods for GM plant viruses

As part of this review, we contacted 35 research groups in 18 countries working with infectious clones of plant viruses, requesting risk assessments, standard operating procedures and details of any permits or licences required by their competent authorities to work with ICs.

Most respondents were United Kingdom or United States based and designated their plant virus IC work as requiring BSL2 and therefore subject to the requirements laid out in Table 1. However, specific containment measures are based on the risk assessment and depend on the nature of the plant virus and the way in which it is being used. The following section therefore details aspects of facility design, equipment and operating procedures specifically tailored to containment of plant virus infectious clones. Examples are drawn from the published literature and information provided by scientists working with plant virus ICs. As many plant viruses have arthropod vectors, strategies are needed not only for containment of infected plant material but also any vectors (Hogenhout *et al*., 2008). More generic guidance on commissioning and building plant quarantine facilities and developing standard operating procedures is available from national competent authorities such as the HSE (UK), the NIH (USA), the Canadian Food Inspection Agency and Adair and Irwin (2008).

### Biological containment methods

Biological containment involves taking steps to render contained organisms biologically incapacitated, and in the context of plant virus infectious clones, this can be achieved in a variety of ways and at a number of stages in the IC construction, modification and inoculation process.

In most cases, plant virus genomes are maintained and propagated in the laboratory within disarmed *Escherichia coli* strains such as DH5α, which are not pathogenic to humans, animals or plants. In addition, eukaryotic promoters are used to reduce the likelihood of the viral genome being transcribed within the prokaryotic bacterial host. This means that minimal physical and chemical containment methods are then required for these bacterial cultures. Similarly, thought should be given to the temporal order of plasmid construction, for example adding the promoter sequence last to delay the point in the development pipeline at which the clone becomes infectious.

The plasmid containing the viral genome is then often transformed into disarmed strains of *A. tumefaciens* such as C58C1 (pMP90) from which the tumour inducing genes have been removed (Wagner *et al*., 2004). However, these strains of *A. tumefaciens* are infectious to plants, and when containing plasmids with genetically modified viral genome sequences, they must be handled accordingly.

When using plant virus ICs as expression vectors, as is the case for molecular farming, the viral genome itself may be modified to be less virulent or transmissible (HSE, 2004; Plchova *et al*., 2017). There are many examples of so‐called deconstructed vector systems, in which a part of the viral genome required for systemic spread is removed and delegated instead to a transgenic host plant (Gleba *et al*., 2007). For example, deletion of the Potato virus X movement protein prevents systemic spread of PVX except in transgenic plants expressing the movement protein (Manske and Schiemann, 2005).

This approach is less useful for studying the nature of plant viruses and their interaction with their hosts, or for germplasm screening, where fully infectious clones are desirable as a source of viral inoculum rather than as a biotechnology tool. For viruses with segmented genomes, some degree of biological containment prior to inoculation may still be achieved by maintaining different parts of the viral genome within different cDNA clones. The host plant then needs to be co‐inoculated with different clones to enable complete infectious virus particles to be created, as demonstrated for the tripartite Barley stripe mosaic virus (BSMV) genome (Lee *et al*., 2012). Separating viral genome components in this way reduces the risk of accidentally releasing the entire viral genome prior to inoculation, but does not reduce the risk posed by the full virus once reconstructed *in planta*.

Biological containment may also be used to prevent or limit the transmission of IC‐derived plant viruses by insect or arthropod vectors. For example, the viral genome may be modified to remove the genes required for transmission, as shown for Tobacco rattle tobravirus transmission by nematode vectors (Hernandez *et al*., 1997). However, this requires knowledge of the genetic components required for vector transmission, which is lacking for most plant viruses. In addition, limiting vector transmission in this way precludes the study of vector transmission itself.

In practice, a more broadly applicable biological method of preventing vector transmission is to conduct experiments, particularly those using vectors, at a time of year when they would not be able to establish outside of the containment facility. Similarly, experiments can be conducted at a time of year when the host plant is not being widely grown. Another approach used for pollen‐ and seed‐transmitted viruses is to prevent flowering or remove flowering plant parts to prevent pollen and seed transmission (Adair and Irwin, 2008; Department of Agriculture, South Africa, 2004).

### Facility design

**Figure 1:**
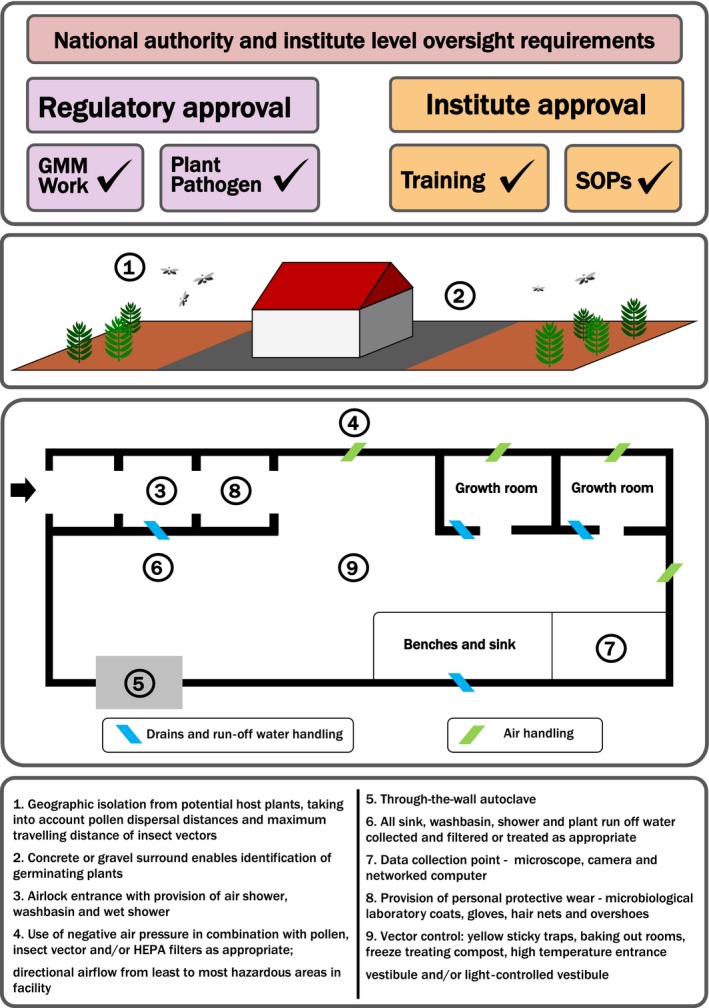
Representative schematic of a containment facility for research involving plant virus infectious clones. Note that specific requirements are determined by individual risk assessments; not every measure will be required or appropriate for a specific virus or use.

Many of the facility considerations for containment of plant viruses are addressed by generic guidance for plant quarantine buildings (see Adair and Irwin, 2008) and subject to the requirements for the designated biosafety level as summarized in Table 1 However, some aspects of facility design are particularly relevant to plant virus containment, as laid out below and in Figure 1. Note that requirements for each aspect of facility design are dependent on risk assessment; not all facility design measures will be required for every IC use.

### Location and surroundings

Geographical isolation from potential hosts is a useful tool for the containment of many plants and plant‐associated microorganisms. In the case of GM crop plants, pollen dispersal distances are often used in risk assessments, particularly for confined field trials where there is limited physical containment. Risk of introgression can be considered low when there are no crops or cross‐pollinating wild relatives within the dispersal distance.

Pollen dispersal distances may also be useful for assessing the risk of escape and establishment of pollen‐transmitted viruses and setting physical, biological and/or chemical containment requirements accordingly. The same applies to identifying the maximum travel distances of insect vectors in the case of vector‐transmitted viruses. In some cases, it may be permissible to relax physical and other containment measures due to geographical isolation.

Laying out the ground immediately surrounding the facility with concrete allows rapid identification and removal of any germinating plants that could act as hosts for contained viruses or virus vectors. This measure may also be useful in the case of seed‐transmitted viruses when the research has involved collection of virus‐infected seed which could germinate and disseminate the virus if released. However, it should be noted that the primary route of seed escape would be on researchers’ clothing and that seeds might not be shed immediately.

### Entrances, exits and vestibules

Air lock entrances with interlocking doors are useful not only for controlling access to the facility via key cards, but also serve as an additional control for highly mobile insect vectors. The vestibule at the entrance to a growth room can be enhanced to prevent vector entry or escape by the use of high temperatures, or controlled lighting so that the vestibule is always darker than the containment room thus discouraging insect egress due to light attraction. Additionally, air lock compartments with powerful air showers can be used to ensure the removal of any insects from clothing and equipment at entry and exit. The United States Department of Agriculture Animal and Plant Inspectorate Service (USDA‐APHIS) recommend full wet showers on exit when working with quarantine insect vectors (USDA‐APHIS 2010).

### Air handling

Many plant growth facilities rely on air conditioning and air circulation systems to maintain appropriate temperature, humidity and CO_2_ levels. Depending on the nature of the virus and the work conducted, suitable filters may be required to trap any potentially infected particulate matter, such as pollen, petals and insects. While HEPA filters are a requirement at BSL3, Phillipson and Weekes (2005) observe that they are unlikely to be necessary for most plant virus work. However, this does not consider the requirements of multipurpose facilities housing various quarantine organisms with diverse containment requirements.

USDA‐APHIS (2010) suggests the use of 80 mesh for working with plant viruses and their vectors. They also suggest directional airflow with movement from the least to most hazardous rooms within a facility.

As previously mentioned, maintenance of negative air pressure compared to the surrounding environment may be unnecessary or even detrimental to the containment of plant viruses and their vectors. However, it may be required for other organisms within the facility. In this case, influx air should be filtered to prevent the ingress of insect vectors due to negative pressure. Air treatment systems should also be designed to accommodate access for repair and routine maintenance without compromising containment.

### Equipment

#### Autoclaves

Autoclaves for inactivation of solid waste are required within the building at BSL2 and within the facility at BSL3. At the two BSL3 plant virus containment facilities we surveyed (UK and Poland), double doored ‘through the wall’ autoclaves are installed to prevent material becoming contaminated after autoclaving but before removal from the facility, exceeding general requirements for this BSL.

As with all decontamination measures, it is important to validate the effectiveness of autoclave run settings (i.e. temperature and run duration) for inactivation of viruses. Temperature indicator strips and electronic monitoring should be used to confirm the correct function of autoclaves.

#### Wastewater treatment

Depending on the risk assessment, it may be necessary to collect and treat some or all wastewater in a facility, such as run‐off from plant watering, washing up basins, hand‐wash sinks and showers. Purpose built wastewater treatment units may be used combining chemical, thermal and UV treatment. However, in some cases, dilution alone may serve to render plant viruses noninfectious. For example, >1/512 dilution of Barley stripe mosaic virus inoculum abolishes infectivity of this virus (Urban *et al*., 2011). Screens or filter socks over drains may be used to collect solid material such as plant waste from run‐off water, which can then be autoclaved (Adair and Irwin, 2008). As many facilities house multiple organisms requiring containment, treatment of common aspects such as water must be validated for all contained organisms, not just viruses (Urban *et al*., 2011).

#### Data collection equipment

Removal of potentially contaminated data collection equipment from containment facilities poses a significant risk of accidental release of plant viruses. The installation of networked computers with associated hardware such as scanners, printers and memory card readers within the facility negates the need to remove laboratory notebooks and cameras, as data can be uploaded to shared drives from within the facility.

### Other facility considerations

It is advisable to use a Class II laminar flow biosafety cabinet for opening packages received from elsewhere, as imported plant material may house other pathogens besides the desired virus strain (USDA‐APHIS 2010). As with any containment facility, there should be an alarm system to detect the failure of critical systems such as loss of negative pressure, as well as alarmed emergency exits. Facilities and equipment should be regularly checked and routinely serviced. Where downstream analysis of infected plant tissue is required, such as molecular characterization or microscopy, there should be provisions for this within the containment facility or in another facility running at the same BSL, with transfer in sealed nonbreakable containers. If this is not possible material, should be inactivated before removal from the facility, for example by harvesting into biocidal lysis buffer.

### Standard operating procedures (SOPs)

Containment of plant viruses relies not only on adequate facilities but also strict adherence to standard operating procedures (SOPs) for facility operations and experiments, designed to ensure both personnel safety and effective containment. As with facilities and equipment, these should be based on a risk assessment and tailored to the work being done, avoiding unnecessary blanket measures and ensuring that all requirements are achievable.

#### Personal protective wear

Personal protective wear refers to garments worn by researchers and facilities staff to protect them from biological or chemical hazards. However, in the case of plant virus containment, such items also reduce the risk of infectious material leaving the facility on clothing, skin or hair.

Microbiological laboratory coats are required as standard in plant containment facilities, and should ideally be kept within the facility and decontaminated prior to removal, or else transported within sealed containers, as contaminated laboratory coats are an obvious source of accidental virus release. For facilities housing growth rooms running at different BSLs, it is advisable to have different coloured laboratory coats associated with each level. Additional laboratory coats or disposable boiler suits should be provided to engineers, depending on the nature of work to be done.

Gloves are required at BSL3 and are subject to risk assessment for BSL2. While gloves may aid in preventing virus spread, it is important to consider when they should be changed or removed to prevent contamination of communal surfaces, such as door handles and computer keyboards. This is particularly relevant to mechanically transmitted viruses which could be transferred to noninfected plants via contaminated surfaces.

As previously described, prevention of flowering or removal of flowering plants is often used as a biological containment measure. However, where experiments require flowering plants infected with viruses transmitted via pollen, and risk assessment dictates that this must be contained, hairnets may be worn to reduce the risk of pollen spread. These are removed and disposed of prior to exiting the containment facility.

While many plant containment facilities use chemical foot baths to decontaminate shoes on exit, an alternative is to use disposable overshoes.

#### Waste and equipment decontamination

The most common means of inactivating biological material is by autoclaving (see previous section under ‘Equipment’). However, if waste or equipment needs to be transported within or between facilities prior to autoclaving, additional steps should be taken to inactivate or contain it. For example, glassware may be soaked in disinfectant prior to removal for autoclaving. Any such protocol must be validated for successful inactivation of the target virus, rather than simply following generic protocols or manufacturers’ instructions.

Chemical decontamination methods should be validated for equipment that cannot be autoclaved, such as plastic plant pots and trays. These methods may need to be effective both against viruses and against insect vectors (see ‘Vector Control’ below). If this cannot be achieved, it may be advisable to use single‐use equipment.

Limits are routinely set on maximum viral inoculum volumes (e.g. <100 mL) to ensure effective disinfection of liquid waste and containment of spills, and to reduce the likelihood of infective doses being present in wastewater, negating the need for wastewater treatment.

As previously outlined under ‘Equipment’, decontamination of run‐off water is dependent on risk assessment and not always required. In facilities without sealed floors (i.e. BSL2 glasshouses), run‐off should be minimized and the ground treated periodically to inactivate virus particles.

#### Surface sterilization

As with waste disposal, surface sterilization products and methods and should be validated for activity against the target organism, for example by swabbing benches after sterilization and inoculating test plants. Protocols for hand washing also need to be appropriate for the viral system being used rather than simply the BSL. For example, quarantine procedures for TMV at Purdue University (USA) specify the use of cows’ milk to wash hands in, particularly for smokers, to inactivate TMV and prevent mechanical transmission. They note that 70% ethanol is not effective (Adair and Irwin, 2008).

In addition, care should be taken to ensure that equipment used throughout a facility, such as watering cans, do not become contaminated, as this could result in accidental transfer of mechanically transmitted viruses between plants during watering. Any such equipment that comes into contact with infected plant material should be decontaminated prior to further use.

#### Vector control

Control of disease vectors within research facilities is a general requirement laid out by regulators in many countries including the United Kingdom and United States. This is particularly pertinent to containment of plant viruses with known or potential native insect vectors. However, such control may be achieved in several different ways.

Many facilities opt for routine and reactive chemical treatments, monitoring for the presence of insect vectors using yellow sticky traps. Good housekeeping and removal of discarded plants and dead leaves are also recommended. Where insects are already present, Adair and Irwin (2008) suggest ‘baking out’ growth rooms at 40 °C for 2–3 days between experiments to kill insects, but advise consideration of whether this will damage equipment. This is also not appropriate for facilities with continuously running experiments.

Alternatively, freezing compost for 48 h at −20 °C prior to use has been found to be effective for excluding arthropod pests from the facility when used in combination with airlock entry and exits, heat trap vestibules and air showers.

The protocols of many BSL2+ facilities also preclude the movement of plants between facilities and the quarantining of any plants coming in from facilities running at a lower BSL. This is because contaminated plants may introduce both insect vectors and wild‐type viruses along with other pathogens that could confound study results. Similarly, where seed‐borne viruses are of concern, the movement of seed may be controlled and only confirmed virus‐free seed used.

Finally, restrictions on personnel movement can help to prevent introduction of vectors. For example, some SOPs state that researchers should not enter plant containment facilities after visiting insectaries or field sites, or participating in recreational activities outdoors.

### Monitoring and training

High‐specification containment facilities and stringent operating procedures mean nothing in the absence of staff compliance. Staff therefore need to be adequately trained in all relevant protocols and understand the rationale behind them. The principal investigator or facilities manager should oversee and assess training and monitor compliance. Many institutes review and update their SOPs annually, and all staff should be involved in this process and kept abreast of any changes.

## Regulation of research involving plant virus ICs

In many countries, plant virus ICs and other plant pathogens require regulation both as genetically modified organisms and as disease‐causing agents, with approval for their use granted by at least one national competent authority and subject to multiple laws regarding both human health and the environment. Contained use activities may also be regulated at both the national and local level. Researchers must therefore ensure that their activities conform to all relevant regulations and that approval has been sought from all competent authorities. Here, we illustrate the complexity of the regulatory and approval process faced by plant virus researchers, focusing on the UK framework with comparisons made to other countries.

### Laws governing contained use of plant virus ICs

In the United Kingdom, the Genetically Modified Organisms (Contained Use) Regulations (HSE 2014) set out the requirement for containment measures when working with genetically modified microorganisms (GMMs) in order to limit risks to both human health and the environment. In addition, the Environmental Protection Act (EPA 1990) sets out the requirement for appropriate measures to “avoid damage to the environment that may arise from escape or release from human control” of GMMs. The UK regulatory framework for the contained use of plant virus ICs as GMMs is therefore guided by these two pieces of legislation.

### Competent authorities overseeing plant virus research

Contained use of GM plant viruses in the United Kingdom is overseen by the Health and Safety Executive (HSE) working with the Department for the Environment, Food and Rural Affairs (Defra) in England and Wales, and Science and Advice for Scottish Agriculture (SASA) in Scotland, with equivalent legislation and oversight in Northern Ireland by the Health and Safety Executive Northern Ireland (HSE‐NI) and the Department for Agriculture, Environment and Rural Affairs (DAERA).

There is a requirement to notify HSE (UK) of all intended contained uses of GMMs. Two separate notifications are required; firstly of the premises to be used (for all BSLs) and secondly of individual contained uses (BSL2‐4). Contained use at BSL3/4 requires consent from the competent authority, rather than simply notification.

### Regulation of plant pathogens

In addition to being GMMs, plant virus ICs may also be subject to additional regulation as plant pathogens. Many countries set and enforce prohibitions on the import, movement and keeping of certain plants and plant pathogens. Such prohibitions generally apply to nonindigenous strains, those subject to an eradication campaign, or those that exhibit increased risks to plant health due to increased pathogenicity, host range or survival. In the United Kingdom, researchers must apply to APHA (an agency of Defra) for a licence to work with prohibited plant viruses, in addition to the previously outlined GM notification to HSE. Detailed descriptions of containment procedures and facilities are required as part of the licence application, and effective containment and destruction of the prohibited virus are a condition of licence approval. Equivalent systems exist in other countries including the United States, Australia and Canada, where prohibited pathogens require a permit for contained use and are therefore referred to as ‘permitted pathogens’ (Australian Government, 2017; CFIA, 2017; USDA‐APHIS, 2016). In all cases, provision of a licence or permit requires inspection and approval of the research establishment by a local and/or national competent authority, which may be distinct from the competent authority overseeing approval for GM work. For example, in Australia, import of plant pests requires approval by the Department of Agriculture and Water Resources, while accreditation of facilities conducting research with GMOs falls to the Office of the Gene Technology Regulator (http://www.ogtr.gov.au/).

## Barriers to meeting plant virus containment requirements

While the guidance set out within this review is theoretically achievable, in some cases barriers to containment of plant virus infectious clones remain.

For example, specifications for facility design assume the reliable supply of utilities such as electricity and water to ensure continuous operation of control systems, supplemental lighting and air conditioning. Such provisions are taken for granted in many countries but may be harder to achieve in countries or regions where utility supplies are unreliable.

Expertise in specific viruses may be primarily in institutes where the host plant is widely researched and grown extensively, thus presenting an increased risk of virus spread within and between growth facilities. Geographical isolation may also not be feasible due to the presence of field trials and commercial plantings in proximity to the containment facility. This is less of an issue for unmodified endemic virus clones, but may increase the risk posed by modifications that alter virus pathogenicity or transmission, or research into exotic viruses of native crops. Biological control methods such as conducting experiments at a time of year when the host and/or vector is absent from the environment along with stringent physical and chemical containment measures may therefore be required.

Barriers to capacity and location may be overcome by international collaborations that facilitate the use of plant virus infectious clones in counties or areas where the plant host and vector are absent. Such examples include the use of infectious clones of Cassava mosaic virus in the United States to screen cassava germplasm for resistance prior to conducting field trials in East Africa (Beyene *et al*., 2016; Kuria *et al*., 2017). This virus is a major threat to cassava production in sub‐Saharan Africa but poses little appreciable risk in the United States where cassava is not cultivated. However, the possibility of host species jumps or unidentified insect vectors should always be considered and appropriate containment measures applied.

The main barrier to successful containment is arguably that posed by human error or failure to comply with SOPs, as demonstrated by containment breaches involving human pathogens (Sample, 2014; Weiss *et al*., 2015). Diligent oversight of staff training, competence and compliance is therefore key to successful plant virus containment. It is also important that all staff understand the rationale for containment measures and the risks associated with a failure in plant virus containment.

## Conclusion

Plant virus ICs are important molecular tools in many areas of biology. However, their status as both GMMs and plant pathogens necessitates their containment by a combination of biological, physical and operational measures to prevent harm to the environment and allay concerns regarding perceived or potential risks to human health. This review is the first to bring together biosafety and regulatory considerations from multiple international sources for working with plant viruses and is therefore a valuable resource for all researchers developing projects involving the use of plant viruses in a range of biotechnology fields.

The appropriate containment strategies for plant virus ICs should be decided based on case by case assessment of the risks posed and the measures needed to mitigate them, rather than assuming that generic containment measures informed by a given BSL will be sufficient. Similarly, all containment methods required should be validated for the IC and operational system in question, rather than assuming their efficacy. Adequate staff training and monitoring of compliance are also essential for effective containment.

Further research on the persistence of inserted DNA constructs along with the relative fitness of modified clones compared to wild‐type viruses would be helpful in aiding the risk assessment process and ensuring that appropriate containment measures are in place. This may vary depending on the virus or modification in question and would require research on a case by case basis.

The notification or approval process for use of plant virus ICs may require two or more applications to various competent authorities to comply with separate regulations governing the use of firstly, GMMs and secondly, plant pathogens, in addition to being regulated at both local and national/state levels. A more streamlined regulatory framework that addresses this dual nature of plant virus ICs and other plant pathogens may save duplication during the application process, both in the United Kingdom and elsewhere.

The generation and use of plant virus infectious clones are no longer the preserve of the plant molecular biologist, having rapidly gained traction in other fields, particularly biomedicine. It is therefore increasingly important that the growing body of researchers using these valuable tools are aware of the potential risks they pose and how to mitigate against them.
